# END USER FEEDBACK TO IMPROVE PATIENT-FOCUSED TELEHEALTH TRAINING

**DOI:** 10.1093/geroni/igae098.1540

**Published:** 2024-12-31

**Authors:** Paul Freddolino

**Affiliations:** Michigan State University, East Lansing, Michigan, United States

## Abstract

After older-adult home-delivered meal recipients in a predominantly white, rural Midwest community received tutoring on basic digital skills including safety and security, email, internet browsing, videoconferencing, and photo tools, they were presented with a five-part patient-focused Telehealth 101 mini-course. Content included types of telehealth (real time encounters, remote patient monitoring, and patient portals), what to expect from providers, how to prepare for telehealth encounters, and a live simulation of a telehealth visit. The telehealth mini course was also offered virtually on Zoom to predominantly black groups at five urban senior centers. While participants rated the sessions positively, and patient engagement in healthcare (Patient Activation Measure scores) increased significantly after the training, critical feedback provided insights into how to improve the training. During 2023 the project team worked with six ‘co-producers’ to get their suggestions and assessments of revisions under consideration for the training materials. Concurrently, revised resources – including two new topics in the telehealth content – were piloted with two predominantly white senior centers and two predominantly black senior centers. Feedback has again been positive and included insights for continuous quality improvement. Our experience shows the contributions and challenges encountered in seeking and engaging end users as co-producers of health-related content.

